# Antimalarial Therapy Selection for Quinolone Resistance among *Escherichia coli* in the Absence of Quinolone Exposure, in Tropical South America

**DOI:** 10.1371/journal.pone.0002727

**Published:** 2008-07-16

**Authors:** Ross J. Davidson, Ian Davis, Barbara M. Willey, Keyro Rizg, Shelly Bolotin, Vanessa Porter, Jane Polsky, Nick Daneman, Allison McGeer, Paul Yang, Dennis Scolnik, Roy Rowsell, Olga Imas, Michael S. Silverman

**Affiliations:** 1 Queen Elizabeth II Health Sciences Centre, Dalhousie University, Halifax, Canada; 2 Dalhousie University, Halifax, Canada; 3 Lakeridge Health Center, Oshawa, Canada; 4 University of Toronto, Toronto, Canada; 5 Toronto Medical Laboratories/Mount Sinai Hospital Department of Microbiology, Mount Sinai Hospital, Toronto, Canada; 6 St. Michael's Hospital Department of Family & Community Medicine, Toronto, Canada; 7 Ontario Ministry of the Environment, Etobicoke, Canada; Cincinnati Children's Hospital Medical Center, United States of America

## Abstract

**Background:**

Bacterial resistance to antibiotics is thought to develop only in the presence of antibiotic pressure. Here we show evidence to suggest that fluoroquinolone resistance in *Escherichia coli* has developed in the absence of fluoroquinolone use.

**Methods:**

Over 4 years, outreach clinic attendees in one moderately remote and five very remote villages in rural Guyana were surveyed for the presence of rectal carriage of ciprofloxacin-resistant Gram-negative bacilli (GNB). Drinking water was tested for the presence of resistant GNB by culture, and the presence of antibacterial agents and chloroquine by HPLC. The development of ciprofloxacin resistance in *E. coli* was examined after serial exposure to chloroquine. Patient and laboratory isolates of *E. coli* resistant to ciprofloxacin were assessed by PCR-sequencing for quinolone-resistance-determining-region (QRDR) mutations.

**Results:**

In the very remote villages, 4.8% of patients carried ciprofloxacin-resistant *E. coli* with QRDR mutations despite no local availability of quinolones. However, there had been extensive local use of chloroquine, with higher prevalence of resistance seen in the villages shortly after a *Plasmodium vivax* epidemic (p<0.01). Antibacterial agents were not found in the drinking water, but chloroquine was demonstrated to be present. Chloroquine was found to inhibit the growth of *E. coli in vitro*. Replica plating demonstrated that 2-step QRDR mutations could be induced in *E. coli* in response to chloroquine.

**Conclusions:**

In these remote communities, the heavy use of chloroquine to treat malaria likely selected for ciprofloxacin resistance in *E. coli*. This may be an important public health problem in malarious areas.

## Introduction

Local patterns of both human and agricultural antibiotic consumption have been shown to correlate with local rates of antimicrobial drug resistance for many antimicrobial-pathogen combinations [1], [2]. Thus, in truly remote communities, with little or no access to antimicrobials and limited interaction with other populations, rates of antimicrobial resistance should be negligible. High rates of fluoroquinolone resistance among Gram-negative bacilli (GNB) have been documented in tropical environments that have extensive use of fluoroquinolones [3]–[5], and have spread from there to the developed world [6]. To our knowledge, widespread fluoroquinolone resistance has not been previously documented to occur in the absence of fluoroquinolone use.

Chloroquine, 7-chloro-4-(4-diethylamino-1methylbutylamino)-quinoline, is extensively used throughout the tropics for its activity against erythrocytic forms of *Plasmodium species*. In Guyana, fever with no obvious anatomic source is empirically treated with chloroquine until malaria smears have been completed. In most malarious countries, chloroquine is still an important component in the treatment of *P. vivax* [7], [8], and despite resistance in *P. falciparum* [8], recurrent empiric treatment with chloroquine several times a year is widespread in the tropics, primarily due to its low cost [9]. In fact national household surveys in 28 African countries have shown that an average of 42% of children under 5 years with fever were treated with an antimalarial, and despite its poor efficacy in African children with *P. falciparum*, more than 80% of these reported treatments were with chloroquine [10]. Chloroquine is also used in the developed world in the treatment of rheumatoid arthritis, and systemic lupus erythematosis [11], [12]. Chloroquine inhibits DNA and RNA polymerase activity *in vitro* [13], halts the replication of *Legionella pneumophila* by limiting the availability of iron necessary for intracellular growth [14], and has weak antibacterial properties [15] against *Salmonella sp.* [16] and *Bacillus subtilis* [17].

**Figure 1:**
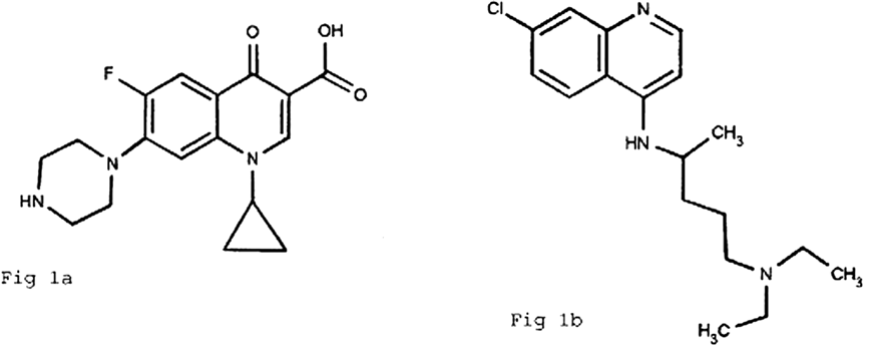
A comparison of the chemical structures of ciprofloxacin (A) and chloroquine (B).

The pharmacore of the fluoroquinolones and chloroquine are similar (Figure 1). In fact the origins of the quinolone class are from the use of chloroquine as an antimalarial [18]. A compound isolated from the commercial preparation of chloroquine was modified to produce the first marketed quinolone, nalidixic acid [18], [19]. Fluorine was subsequently added to produce the fluoroquinolones, resulting in both an increase in potency and spectrum. We hypothesized that the extensive use of chloroquine in tropical countries could select for fluoroquinolone resistance among *E. coli* despite the absence of fluoroquinolone use.

## Results

### Surveillance cultures

A total of 535 rectal swabs were obtained: 240 in 2002, 152 in 2003 and 143 in 2005 (Table 1). Only 15.4% of individuals reported antibiotic exposure in the previous five years. No patients received antibiotics within 30 days of sampling. All participants denied fluoroquinolone use. In 2003, 30% reported chloroquine use within the previous 6 months and in 2005, 86% reported chloroquine use within the previous 2 years.

**Table 1:** Demographics of the study population.

Demographics	2002	2003	2005	Total
Number of patients screened	240	152	143	535
Age Group				
Children <17 years (%)	105 (43.8)	63 (41.4)	57 (39.8)	225 (42.1)
Adults 17–65 years (%)	124 (51.7)	75 (49.3)	76 (53.3)	275 (51.4)
Elderly >65 years (%)	11 (4.6)	14 (9.2)	10 (6.9)	35 (6.5)
Sex				
Number of males (%)	98 (40.8)	72 (47.5)	62 (43.6)	232 (43.3)
Number of females (%)	142 (59.2)	80 (52.5)	81 (56.6)	302 (56.4)
Village of residence (%)				
Bartica	0	24.8	7.7	10.8
Remote villages				
Jawalla	6.0	9.2	17.5	10.9
Kako	25.0	24.1	6.3	18.5
Kamarang	23.9	41.8	19.6	28.4
Phillipai	27.7	0	1.4	9.6
Waramadong	17.4	0	42.6	20
Quebanang	0	0	5.5	1.8
Fluoroquinolone resistant *E.coli* (%)	8 (3.8)	16 (10.2)	5 (3.5)	5.4
Prior antibiotic use (%)	23 (9.6)	28 (18.4)	26 (18.1)	15.4
Prior chloroquine use (%)	N/A	30.2*	86**	NA

N/A = not available/not applicable.

* chloroquine use within the previous 6 months.

** chloroquine use within the previous 2 years.

A total of 30 ciprofloxacin-resistant *E. coli* isolates were isolated from specimens of 29 (5.4%) of the 535 participants (Table 1 and 2) (one patient in 2005 carried 2 different isolates), and were from children (12 of 225 [5.3%]) and adults (17 of 310 [5.5%]) with an age range of 8 months to 65 years. Resistant isolates were obtained throughout the study period [8 of 240 (3.8%) in 2002, 16 of 152 (10.2%) in 2003, and 5 of 143 (3.5%) in 2005.

**Table 2:** Characteristics of ciprofloxacin-resistant *E. coli* isolated from Guyana.

Strain No.	Year	Village	Ciprofloxacin MIC (µg/mL)	Chloroquine MIC (µg/mL)	*gyrA*	*gyrB*	*parC*	*parE*	*XbaI* PFGE type
37	2002	Waramadong	2	2500	S83L	–	S80I	–	I
60	2002	Waramadong	>32	20,000	S83L/D87N	–	S80I		J
68	2002	Waramadong	>32	10,000	S83L/D87N	–	S80I		J
110	2002	Kako	>32	80,000	S83L/D87N	–	S80I	–	G
119	2002	Kako	>32	10,000	S83L/D87N	–	S80I	S458A	L
195	2002	Kamarang	>32	20,000	S83L/D87N	–	S80I/E84G	–	G
212	2002	Jawalla	6	5,000	S83L/D87N	–	S80I	–	M
223	2002	Kamarang	>32	20,000	S83L/D87N	–	S80I	S458T	N
257	2003	Kamarang	>32	80,000	S83L/D87N	–	S80I/E84V	–	A
261	2003	Kamarang	>32	10,000	S83L/D87N	–	S80I	S458A	C
263	2003	Kamarang	>32	10,000	S83L/D87N	–	S80I	S458A	O
268	2003	Kamarang	>32	20,000	S83L/D87N	–	S80I	S458A	O
287	2003	Kamarang	>32	40,000	S83L/D87N	–	S80I	–	E
296	2003	Kamarang	>32	20,000	S83L/D87N	–	S80I	S458A	P
308	2003	Kamarang	>32	20,000	S83L/D87N	–	S80I	L416F	T
312	2003	Kako	>32	10,000	S83L/D87N	–	S80I	S458A	E
324	2003	Kako	>32	5,000	S83L/D87N	–	S80I	–	H
329	2003	Kako	>32	80,000	S83L/D87N	–	S80I	S458A	P
339	2003	Kako	>32	20,000	S83L/D87N	–	S80I	S458A	P
362	2003	Bartica	>32	10,000	S83L/D87N	–	S80I	–	Q
363	2003	Bartica	>32	40,000	S83L/D87N	–	S80I	S458A	R
369	2003	Bartica	16	5,000	S83L/D87N	–	S80I	–	D
371	2003	Bartica	>32	40,000	S83L/D87N	–	S80I	S458A	S
378	2003	Bartica	>32	40,000	S83L/D87N	–	S80I	S458A	X
47	2005	Waramadong	4	10,000	S83L	–	S80I	–	K
76	2005	Jawalla	>32	20,000	S83L/D87N	–	S80I	–	B
86	2005	Bartica	>32	20,000	S83L/D87N	–	S80I	S458A	Y
97	2005	Waramadong	>32	40,000	S83L/D87N	–	S80I	S458A	C
103	2005	Waramadong	>32	10,000	S83L/D87N	–	S80I	–	Z
119	2005	Jawalla	>32	20,000	S83L	–	S80I	S458A	U

The ciprofloxacin MIC ranged between 2 and >32 µg/mL, with 26 of 30 *E. coli* having MIC of >32 µg/mL (Table 2). The chloroquine MIC ranged between 5,000 and 80,000 µg/mL. By contrast, the chloroquine MIC of susceptible *E. coli* isolated from Guyana and Canada ranged between 625 and 1,200 µg/mL. PFGE typing identified 24 distinct strains among the 30 isolates (Table 2).

All ciprofloxacin resistant isolates had mutations in *parC* and *gyrA* commonly associated with fluoroquinolone resistance (Table 2). No *qnrA/B* genes and no evidence of an active efflux system were identified. No QRDR mutations were found among ciprofloxacin-susceptible *E. coli* isolated from the same patient population.

### Clinical correlates of ciprofloxacin resistance

#### Non-Bartica village results

4.8% of patients in the non-Bartica villages carried ciprofloxacin-resistant *E. coli*. In the non-Bartica villages (but not in Bartica) there was a dramatic increase in number of *P. vivax* cases between May and December 2002. The incidence of *P. vivax* rose from a baseline of 4.97 cases/1000 persons/month (standard deviation [SD] = 2.4/1000) to 68.0 cases/1000 persons/month (SD = 23.4/1000) an increase of 1,270% (2 tailed p value<0.0001) (CI for the change 57.3–68.7/1000 persons/month). During the course of this severe epidemic, 1,360 smear confirmed cases of *P. vivax* were reported in the local population of 4,362 persons. Total malaria cases (*P. vivax*+*P. falciparum*) rose from 7.37/1000 persons/month to 74.9/1000 persons/month (p<0.0001) [a 916% increase]. This increase in total cases reflects the increased incidence of *P. vivax*, as there was no significant increase in *P. falciparum* cases at this time. Local treatment for *P. vivax* consists of chloroquine and primaquine. Therefore there was a marked increase in chloroquine use in these communities during this epidemic.

Multivariable analysis that included only the non-Bartica samples, revealed year of testing as the only factor significantly associated with the carriage of ciprofloxacin resistant *E. coli* adjusted for age, sex, and previous antibiotic use. This adjusted analysis demonstrated that among residents of the non-Bartica villages (n = 480), testing shortly after the *P. vivax* epidemic in February 2003 (prevalence of resistance [POR] = 11/108, 10.2%) compared to the non epidemic periods of February 2002 (POR = 8/240, 3.3%) and February 2005 (POR = 4/132, 3.0%) was significantly associated with a higher risk of ciprofloxacin resistance (OR = 3.83, 95% CI: 1.56–9.40, p = 0.003). Similarly, the crude estimate (unadjusted analysis using only non-Bartica samples) demonstrated testing in February 2003 compared to the non epidemic periods of February 2002 and Feb 2005 was still significantly associated with a higher risk of ciprofloxacin resistance (OR = 3.40, 95%CI:1.46–7.95, p = 0.0047).

Crude (unadjusted) analysis including only the non-Bartica villages did not demonstrate age, sex, previous antibiotic use or reported chloroquine use (recent or ever) to be associated with carriage of ciprofloxacin resistant *E. coli*.

#### General analysis results

In the following analyses all patients (including Bartica patients) were included. In combined year analysis, all independent variables (i.e. age, sex, residence in Bartica, year of testing, chloroquine use, use of any anti-malarials, prior antibiotic use) were assessed for association with ciprofloxacin-resistant *E. coli* in multivariable logistic regression analysis. In analysis using variables collected across all three years (age, sex, prior antibiotic use, village of residence, year of testing), testing in 2003 (compared to 2002 and 2005 combined), with adjustment for sex, ever antibiotic use and residence in Bartica, was the only factor significantly associated with risk of ciprofloxacin-resistant *E. coli* carriage (OR = 2.73, 95% CI: 1.16–6.38, p = 0.02). None of the other variables (age, sex, chloroquine use, any-antimalarial use, residence in Bartica, or previous antibiotic use) were associated with ciprofloxacin-resistant *E. coli* carriage.

A crude (unadjusted) analysis also did not demonstrate any association between individual reported chloroquine use (ever, or within the past 6 months) or previous antibiotic use, and carriage of ciprofloxacin resistant *E. coli*. There was no association between use of traditional medicinal herbs, or cassava ingestion with quinolone resistance.

### Water sample analyses

One of the 24 water samples collected in 2004 yielded a single isolate of ciprofloxacin-resistant *E. coli* (MIC of 32 µg/mL) isolated from Bartica river water. High performance liquid chromatography/electrospray ionization/tandem mass spectrometry (LC/MS-MS) demonstrated that one of 13 samples collected in 2005, river water from Kamarang, was found to contain chloroquine at a concentration of 4.7 ng/L. Ciprofloxacin was not detected in any sample. No antimicrobial activity was detected by microbial inhibition testing (MIT) in the 13 water samples tested.

### Chloroquine selection of fluoroquinolone resistance *in vitro*

After serial exposure to chloroquine, three isolates were able to grow in the presence of ciprofloxacin at 0.032 µg/mL (Table 3). The ciprofloxacin MIC increased from 0.008 to 0.094 µg/mL in one isolate and to 0.12 µg/mL in two isolates. Sequencing of the QRDR regions of *gyrA* and *parC* revealed an aspartate-87-tyrosine mutation in *gyrA*. Serial exposure to increasing concentrations of chloroquine resulted in a further increase in the ciprofloxacin MIC from 0.12 to 6 µg/mL in two of the three strains. Analysis of *parC* and *gyrA* revealed a second mutation in the QRDR of *parC* resulting in a change from serine-80-isoleucine. No mutations in *gyrB* or *parE* were detected.

**Table 3:** *In-vitro* selection of ciprofloxacin resistance after exposure to chloroquine.

Strain no.	Chloroquine	Ciprofloxacin	*gyrA*	*gyrB*	*parC*	*parE*
	µg/mL	µg/mL				
271–2029^+^	625	0.008	Wild type	Wild type	Wild type	Wild type
271–2029*	5000	0.094	D87Y	–	–	–
270–3118^+^	625	0.008	Wild type	Wild type	Wild type	Wild type
270–3118*	2500	0.12	D87Y	–	–	–
270–3118**	10,000	6.0	D87Y	–	S80I	–
290–9211^++^	625	0.008	Wild type	Wild type	Wild type	Wild type
290–9211*	2500	0.12	D87Y	–	–	–
290–9211**	12,000	6.0	D87Y	–	S80I	–

* 1^st^ step selection after exposure to chloroquine.

** 2^nd^ step selection after exposure to chloroquine.

## Discussion

To our knowledge this is the first report implicating quinoline antimalarial use in the development of bacterial quinolone resistance in the tropics. The resistance mutations that were found in the Guyanese samples, and selected *in vitro* by chloroquine exposure, result in cross resistance to all quinolone antibiotics (including nalidixic acid, norfloxacin, ciprofloxacin, levofloxacin, and moxifloxacin).

Antibiotic resistance has become a global crisis as a result of widespread use of antibiotics in medical and agricultural practice [3]–[5]. However, in the remote Amerindian communities of Guyana there is limited access to antimicrobials. Moreover, there are geographical barriers limiting travel in and out of this region. The villages are located in the Guyana highlands, surrounded by the Pakaraima Mountains and dense tropical rainforest. The villages themselves are at significant elevation (Kamarang is at 1621 ft). One therefore requires air travel to enter this area (143.8 mile flight from Bartica), and then boat transport between communities. These are the headwaters of the Mazzaruni River, and so contamination of water from higher up in the river does not occur. This geographic isolation and thus difficulty with travel leads to financial barriers preventing inhabitants from venturing beyond the region, and also preventing outsiders from venturing in. Furthermore as reserved Amerindian communities, there are political limitations preventing outsiders from travelling into the region [20]–[22]. Whether these communities will be able to remain separate and maintain their culture is unclear due to the fact that mining companies are now hoping to enter this area [23]. Due to the isolation of these communities during the course of our study however, it would be expected that the Amerindians would be subject to negligible rates of antimicrobial resistance. Despite the lack of quinolone exposure, we found faecal carriage of *E. coli* resistant to ciprofloxacin in 5.4% of this population. A large intensive care unit survey in the USA done at the same time as our study, found only 4% of *E. coli* resistant to ciprofloxacin [24].

We cannot with certainty dismiss the possibility that a few individuals traveling outside of the region may have been exposed to fluoroquinolones, and on return, transmitted resistant organisms to their neighbours. However, this hypothesis is unlikely not only because of the isolation of these communities, and thus the rarity of outside travel, but also because the resistant *E. coli* were primarily polyclonal and the strains were widely distributed among inhabitants of different villages. The fact that 24 different clones of quinolone resistant *E. coli* were found is evidence that an occasional traveler would be an unlikely source of the multiple resistant strains and therefore, there must be a local factor selecting for resistance.

Detailed demographic data on the involved communities, and regarding the patients presenting to the clinics, and those tested, are presented in table 1, as well as Appendix S1, Appendix S2, Appendix S3 and Appendix S4. The data suggests that the most common reason for presentation to the clinics was feeling well but wishing a check-up (35%). Fifty three per cent presented with one of the three most common reasons (check-up, musculoskeletal pains, or request for family planning). In fact 82% of patients did not have any infective diagnosis, and only 16% had a bacterial etiology suspected. Furthermore, none of the patients in our study had received an antibacterial antibiotic within 30 days of presentation to the clinic. Therefore we believe that our data reflect the prevalence of quinolone resistant *E. coli* in the general population.

Chloroquine is a weakly active antibacterial with a long half life (4 days) that is given frequently and excreted through the bowel; all factors that may optimize the opportunities to select for bacterial resistance. In this study, laboratory experiments were able to demonstrate that chloroquine exposure in-vitro can select for QRDR mutations in *E. coli*. Unlike quinine, chloroquine is not felt to have a natural counterpart. We suspect that chloroquine found in the drinking water in Kamarang may be the result of contamination by human waste. This suggests widespread exposure to chloroquine, even in the absence of individual malaria treatment. It is also notable that chloroquine use within two years of testing was almost universal and thus identification of an association between chloroquine use and ciprofloxacin resistance within individuals was not possible. Nevertheless, resistance in *E. coli* was highest in the villages that had recently experienced a dramatic *P. vivax* outbreak, and the corresponding increase in chloroquine use may have led to an increased prevalence of ciprofloxacin-resistant *E. coli*. Specific data regarding total chloroquine use in these communities is not available. However we inferred a dramatic increase in use in late 2002, as chloroquine is used for the initial treatment of all cases of suspect malaria until smears are available, and is then continued for confirmed cases *P. vivax*. Therefore the 1,270% rise in *P. vivax* and the 916% increase in total malaria cases during the epidemic period of late 2002, implies a dramatic increase in chloroquine use at that time. It is notable that this data consists of the reported incidence of smear positive and thus TREATED cases, (not including estimates of undiagnosed cases) and therefore does reflect actual chloroquine use.

Chloroquine levels were measured in the water in a non-epidemic year (2005) and thus may have been higher at other times. Although the levels we detected are unlikely to select for fluoroquinolone resistance, detection of chloroquine in the river, likely as a result of human waste, may be an indirect measure of the high volume of chloroquine use in the region. As traditional dosing for *P. vivax* used by the medics includes chloroquine 1 g PO then 500 mg PO 6 hours later, and then 500 mg PO daily×2 more days, very high concentrations are achieved in the upper gut, and these concentrations would exceed those which were used in vitro to generate resistance. A gradient of concentrations would likely be generated throughout the intestines and this may facilitate the emergence of resistance. It is possible that the presence of small concentrations of chloroquine in the drinking water may have provided an ongoing selective advantage to ciprofloxacin resistant *E. coli* within patient's intestines, prolonging carriage.

Some local residents may have acquired quinolone resistant *E. coli* from exposure to contaminated drinking water. Nevertheless, the identification of 24 distinct isolates of quinolone resistant *E. coli* in this population, suggests that a single point source of water contamination would not be able to explain the multiple circulating strains. Rather generation of resistant strains is likely occurring in many different patients' intestines, with some horizontal transmission within the community via drinking water contamination.

The potential exposure to both quinolone resistant organisms and chloroquine itself from human waste contaminated river water may explain the failure to identify an association between individual reported use of chloroquine and carriage of quinolone resistant *E. coli*. Furthermore the reliability of individual reports of chloroquine exposure is unknown. Nevertheless the fact that increased total community chloroquine use (as inferred by testing in a *P. vivax* epidemic year versus non-epidemic years) was significantly associated with prevalence of resistance, is strong ecological evidence of the association of quinoline use with the development of quinolone resistance in the community. This situation is analogous to that seen with antibacterial antibiotics, where there are very few reports of individual use being associated with the development of resistance, but the evidence of total community use leading to increased resistance is more compelling [1], [2]. Indeed, this may be one circumstance in which ecological data are more useful than individual-level data.

Although no clinical outcomes were impacted in this area by quinolone resistance, (due to the lack of local quinolone use), we believe the same phenomenon is likely also occurring in other malarious communities, and may be leading to a lack of clinical efficacy of quinolones. This site was particularly interesting to study, because the lack of the confounding presence of quinolones in the community allowed us to identify the relationship between community wide quinoline use for malaria and quinolone resistance in bacteria.

One limitation of this study is that we failed to rule out the possibility that other quinoline antimalarials, particularly primaquine, which together with chloroquine is the standard treatment regimen in Guyana for cases of *P. vivax*, may also be selecting for ciprofloxacin resistance. Other quinoline and quinoline-like compounds include quinine, mefloquine, quinidine, halofantrine, lumefantrine, piperaquine, pyronaridine, and amodiaquine. Other study limitations include a limited number and timing of water samples, and failure to ask about chloroquine exposure in the first year.

This study is the first to identify human carriage of quinolone resistant organisms in communities that lack any quinolone exposure. It is also the first to identify antimalarial therapy as a factor in the development of carriage of quinolone resistant bacteria. We have reported the selection of *E. coli* resistance by chloroquine in this study. A recent report demonstrated that fluoroquinolone resistance may result in an increase in mortality from *E. coli* infections due to the increased likelihood of inadequate empirical antimicrobial therapy [25]. A similar phenomenon likely also occurs in other organisms treated empirically with quinolones. Indeed persons in these very remote Amerindian Guyanese communities were found to carry multiple other quinolone resistant gram negative organisms including *Salmonella* (data not shown). Quinolone antibiotics have an important role in the therapy of many tropical infectious diseases such as typhoid fever, non-typhoidal *Salmonella*, *Shigella* and enterotoxigenic *E. coli* infections. They also are important in both the tropics and in temperate regions in the treatment of gonorrhoea, urinary tract infections, upper and lower respiratory tract infections, are becoming increasingly important in the therapy of tuberculosis, and have widespread use in hospitalized patients. These antibiotics have excellent oral bioavailability, and do not require refrigeration, making them a critical component of our antibacterial armamentarium in the tropics, and for these reasons the development of resistance to these agents has important consequences. As chloroquine use is widespread throughout the tropics our findings may represent an important public health problem. In fact, although *P. falciparum* chloroquine resistance is widespread, use of quinoline antimalarials will likely continue, and may increase, as they are used in artemisinin combination therapy (ACT) [26]. The large scale recent introduction of these therapies, especially as presently contemplated for use in mass intermittent presumptive therapy (IPT) for population wide malaria control [26], could have a significant impact on the antibacterial efficacy of quinolones.

In light of the large number of lives saved through proper malaria treatment/control, our data does not imply that quinolines should be discontinued from use. However we plan to carry out further studies is to identify whether some quinolines may be less likely to induce quinolone resistance than others, and thus be safer for malaria control programs. Our data also implies that further investigations regarding the efficacy of quinolone antibiotics in other regions of high malarial endemicity are necessary, particularly since many of these areas presently lack facilities for bacterial culture and sensitivity testing, and thus rely on empiric treatment. Furthermore our data adds impetus to programs that lead to the primary prevention of malaria, such as vector control, and the development of vaccine programs, in order to reduce the need for widespread quinoline chemotherapy.

## Methods

### Setting

This study was conducted in the isolated Amerindian communities of north-western Guyana, and the less isolated, ethnically-mixed Guyanese community of Bartica. Access to the Amerindian communities is prohibited to non-Amerindians and requires travel by bush airplane and canoe. These communities have no pharmacy, and only amoxicillin, TMP/SMX and the antimalarials chloroquine, quinine, primaquine, and doxycycline are available via local medics. Interviews with these medics confirm that quinolones (including non-fluorinated quinolones) had never been used in this region. All goods imported to these areas must be registered with the local Amerindian police. Commercial fertilizers and animal supplements are not available.

Outreach clinics visited these villages for 2 weeks in February of 2002, 2003 and 2005. In 2002, visits were made to Jawalla, Kako, Kamarang, Phillipai and Waramadong; in 2003 Jawalla, Kako, Kamarang and Bartica were visited. In 2005, visits were made to Jawalla, Kako, Kamarang, Waramadong, Quebanang and Bartica. All villages except Bartica are highly isolated. These isolated communities are located along the Upper Mazarruni River, and their drinking and river water is from the Guyanese Highlands, and not from any other populated centres. In contrast to the other communities, Bartica can be reached by speedboat, and has 2 physicians who have occasional access to fluoroquinolone antibiotics. Therefore the majority of our analysis was focused on only the highly remote communities.

### Specimen Collection and Transport

Rectal swabs were obtained from consenting individuals presenting to the clinics, who also provided demographic and medical information by interview. Swabs were stored in Amies transport media at ambient temperature during transport to Canada. In 2004, 24 water samples of 200 ml each were collected including six tap and four river water samples from Bartica, three samples from the Kamarang river and one tap water sample from Kamarang village, three samples of river water each from Waramadong village and from the Upper Mazaruni River at Jawalla, and two each from Kako village and Phillipai. In 2005, two river water samples (200 ml each) were collected from each of the remote communities visited as well as one tap water sample from Kamarang. Samples were kept refrigerated during transport.

### Background Clinical Information

In addition to basic demographic information (age, sex, village), individuals who had rectal swabs sampled were asked the following questions.

1.
  - Have you ever taken any antibiotics?
  - If the answer was *no* then the following question was asked: Have you taken any medicines for infection or sickness?
  - If the answer was *yes* the following questions were asked: What antibiotics have you taken? If patients could not recall the names, then choices of available medications (amoxicillin/septrin/metronidazole/doxycyline as well as ciprofloxacin/“cipro”[which is not locally available]) and the colours of these medications (matched with the medics' supplies) were all offered.
  - When did you take them?
2.
  - Have you taken any “bush medicines” (the local term for traditional medicinal herbs)?
  - If yes, what were they?
3.
  - Do you eat Casava (a local staple potato)?
  - Do you drink it fermented (“Kasrik”?)
  - Do you eat cassava bread?
  - If yes, how many times a day do you eat it?
4.  In 2003 patients were also asked:
5.
  - Have you had malaria in the past 6 months?
  - Have you had vivax or falciparum malaria or both?
  - Did you receive ia) chloroquine iib) primaquine iiic) quinine iv d) doxycycline? (each of these medications colors were was also described to assist memory)?
6.  In 2005 Patients were also asked:
7.
  - Have you had malaria in the past 5 years? Ever?
  - How many times have you been treated for malaria?
  - When was the last time you were treated for malaria?
  - The last time you were treated, did you have vivax or falciparum or both?
  - Did you receive chloroquine/primaquine/quinine/doxycycline? (each of these medications colours were was also described to assist memory)?

### Clinical Information Regarding Health Seeking Behaviour of the Studied Communities

Data on the first 501 patients visiting the clinic in 2005 was reviewed. Specific identifying patient data was purged prior to review. Non-nominal demographic data (age, sex, village, as well as attending physician diagnosis recorded on the clinic chart at the end of the patients visit) was tabulated.

### Isolation and identification of fluoroquinolone resistant *E. coli*

On receipt in Canada, rectal swabs were incubated in brain heart infusion broth at 35°C for 24 h and then cultured onto MacConkey agar with and without 1 µg/ml ciprofloxacin and incubated aerobically at 35°C for 24 h. After incubation, Gram-negative bacilli growing on the ciprofloxacin containing plate were identified to the species level by the Vitek GNI card (BioMerieux, Hazelwood, MO). Ciprofloxacin resistance in *E. coli* was defined as an MIC ≥2 µg/mL. Water samples from 2004 were cultured by filtering 50 mL through a sterile 0.45 µm millipore filter and incubating the filter in 20 mL brain heart infusion broth overnight at 35°C. Turbid broths were sub-cultured by spreading a 100 µL aliquot onto sheep blood (5%) and MacConkey agar, with a 5 µg ciprofloxacin disk placed onto the inoculated area. Growth of Gram-negative bacilli within the expected zone of inhibition of the ciprofloxacin disk was streaked to MacConkey agar to obtain a pure culture.

Broth microdilution susceptibilities were performed for all *Enterobacteriaceae* growing on the ciprofloxacin containing plate, or within the ciprofloxacin zone of inhibition using Vitek GNS cards (BioMerieux), with confirmation of ciprofloxacin MICs by Etest® (AB Biodisk).

### Characterization of fluoroquinolone-resistant *E. coli* and fluoroquinolone susceptible control strains

The QRDR regions of the *gyrA*, *gyrB*, *parC* and *parE* genes were amplified as previously described [27], [28]. The presence of *qnr* genes were examined as described by Jacoby *et al* [29]. Purified DNA amplicons were sequenced using a Beckman CEQ8000 capillary sequencer. To determine if energy dependent efflux was contributing to fluoroquinolone resistance, ciprofloxacin MICs were measured in the absence and presence of 10 µg/ml reserpine [30]. A four-fold reduction in MIC in the presence of reserpine was considered evidence of efflux-mediated resistance. Isolates were subject to *Xba*I PFGE [31], [32], and typed using the criteria of Tenover et al [33]. Chloroquine MICs were determined using broth microdilution [34].

### Investigation for potential presence of antimicrobials in water samples

High performance liquid chromatography/electrospray ionization/tandem mass spectrometry (LC/MS-MS) was utilized to analyze the water samples for the presence of 23 different antimicrobials including ciprofloxacin and chloroquine [35]. Microbial inhibition testing (MIT) for the detection of antimicrobial activity in water was carried out using both *Micrococcus luteus* and *Bacillus subtilis* in agar plates and 2 mL of penase treated water samples [36].

### Chloroquine selection of fluoroquinolone resistance *in vitro*

One fluoroquinolone-susceptible *E. coli* obtained from Guyana and two Canadian strains (clinical isolates) from the Queen Elizabeth II Health Sciences Centre, Halifax, Nova Scotia were screened for *gyrA/gyrB* and *parC/parE* mutations, efflux, and *qnrA/B*, then inoculated into100 mL Mueller-Hinton broth (MHB) containing 625 µg/ml of chloroquine and incubated for 24 hours at 35°C. A 100 µl aliquot was removed and sub-cultured into MHB containing 1875 µg/ml chloroquine and incubated for 24 hours at 35°C. The inoculum was then replica plated onto another MHA plate containing 0.032 µg/ml ciprofloxacin. Both plates were incubated for 24 hours at 35°C. When ciprofloxacin containing plates displayed visible growth, colonies on the antibiotic free replica plates were isolated and tested by examining their susceptibility to ciprofloxacin and chloroquine, and by amplification and sequencing of the *gyrA/gyrB* and *parC/parE* genes.

First step mutants, selected with chloroquine, were re-exposed to increasing concentrations of chloroquine in MHB, (up to 16,500 µg/ml), sub-cultured onto MHA containing a corresponding concentration of chloroquine, and replica plated onto MHA with and without 2 µg/ml of ciprofloxacin. As above, colonies growing on the ciprofloxacin containing plates had their coordinate colonies on the ciprofloxacin free media tested by examining their susceptibility to ciprofloxacin and chloroquine, and by amplification and sequencing of the *gyrA/gyrB* and *parC/parE* genes

### Statistical Analyses

All statistical analyses were conducted in SAS 9.1.3 (SAS Institute Inc., Cary, NC). Odds ratios (OR) and their corresponding 95% confidence intervals (CI) were calculated using unconditional multiple logistic regression to identify associations between each potential risk factor and presence of ciprofloxacin-resistant *E. coli* in the sample.

### Ethics Approval

Approval to perform surveillance screening for faecal carriage of antimicrobial-resistant bacteria was obtained from the Research Ethics Board of the Lakeridge Health Corporation, Oshawa, Ontario, Canada. Permission to perform the study was also obtained from the local Amerindian community leaders and the Guyanese government. Verbal informed consent was obtained from each patient, or for the children, from their guardian.

## Supporting Information

Appendix S1 Demographic data regarding the studied villages
(0.03 MB DOC)

Appendix S2 Age distribution of patients presenting to the clinic. First 501 patients 2005
(0.03 MB DOC)

Appendix S3 Review of the reason for presentation of the first 501 patients presenting to clinic in 2005
(0.03 MB DOC)

Appendix S4 Review of the reason for presentation of the first 501 patients presenting to clinic in 2005 by diagnostic group
(0.02 MB DOC)
