# Supplementary material for: Antimalarial Therapy Selection for Quinolone Resistance among Escherichia coli in the Absence of Quinolone Exposure, in Tropical South America
Source: PLoS One. 2008 Jul 16;3(7):e2727. doi: 10.1371/journal.pone.0002727 (PMC2481278; doi:10.1371/journal.pone.0002727)
Supplement: Appendix S1 — Demographic data regarding the studied villages (0.03 MB DOC) [file pone.0002727.s001.doc]

| **Name** | **Population**  **(2006 census)** | **Male** | **Female** |
| --- | --- | --- | --- |
| **Kamarang** | 618 | 316 | 302 |
| **Waramadong** | 778 | 384 | 394 |
| **Kako** | 586 | 308 | 278 |
| **Quebenang** | 226 | 101 | 125 |
| **Jawalla** | 1,043 | 538 | 505 |
| **Phillipai** | 1,111 | 578 | 533 |
| **Bartica** | 11,159 | N/A | N/A |

N/A= not available
