# Supplementary material for: Antimalarial Therapy Selection for Quinolone Resistance among Escherichia coli in the Absence of Quinolone Exposure, in Tropical South America
Source: PLoS One. 2008 Jul 16;3(7):e2727. doi: 10.1371/journal.pone.0002727 (PMC2481278; doi:10.1371/journal.pone.0002727)
Supplement: Appendix S2 — Age distribution of patients presenting to the clinic. First 501 patients 2005 (0.03 MB DOC) [file pone.0002727.s002.doc]

| **Age (years)** | **Count** | **%** |
| --- | --- | --- |
| 0-5 | 93 | 20.53 |
| 6-10 | 76 | 16.78 |
| 11-15 | 78 | 17.22 |
| 16-18 | 16 | 3.53 |
| 19-25 | 35 | 7.73 |
| 26-30 | 33 | 7.28 |
| 31-40 | 46 | 10.15 |
| 41-50 | 41 | 9.05 |
| 51-60 | 22 | 4.86 |
| 61+ | 13 | 2.87 |
| Unknown | 4 |  |
