# Supplementary material for: Antimalarial Therapy Selection for Quinolone Resistance among Escherichia coli in the Absence of Quinolone Exposure, in Tropical South America
Source: PLoS One. 2008 Jul 16;3(7):e2727. doi: 10.1371/journal.pone.0002727 (PMC2481278; doi:10.1371/journal.pone.0002727)
Supplement: Appendix S3 — Review of the reason for presentation of the first 501 patients presenting to clinic in 2005 (0.03 MB DOC) [file pone.0002727.s003.doc]

Note: UTI = Urinary Tract Infection, STI = Sexually Transmitted Infection.
