# Supplementary material for: Antimalarial Therapy Selection for Quinolone Resistance among Escherichia coli in the Absence of Quinolone Exposure, in Tropical South America
Source: PLoS One. 2008 Jul 16;3(7):e2727. doi: 10.1371/journal.pone.0002727 (PMC2481278; doi:10.1371/journal.pone.0002727)
Supplement: Appendix S4 — Review of the reason for presentation of the first 501 patients presenting to clinic in 2005 by diagnostic group (0.02 MB DOC) [file pone.0002727.s004.doc]

Group A= Non-infectious etiologies

Group B= Infectious etiologies, likely bacterial

Group C= Infectious etiologies, likely viral

Note: Totals exceed 501 as some patients presented with more than 1 diagnosis
